# Supplementary material for: Systemic Analyses of Cuproptosis-Related lncRNAs in Pancreatic Adenocarcinoma, with a Focus on the Molecular Mechanism of LINC00853
Source: Int J Mol Sci. 2023 Apr 27;24(9):7923. doi: 10.3390/ijms24097923 (PMC10177970; doi:10.3390/ijms24097923)
Supplement: Supplementary file 1 [file ijms-24-07923-s001.zip › Supplementary Figures S1-S8.pdf]

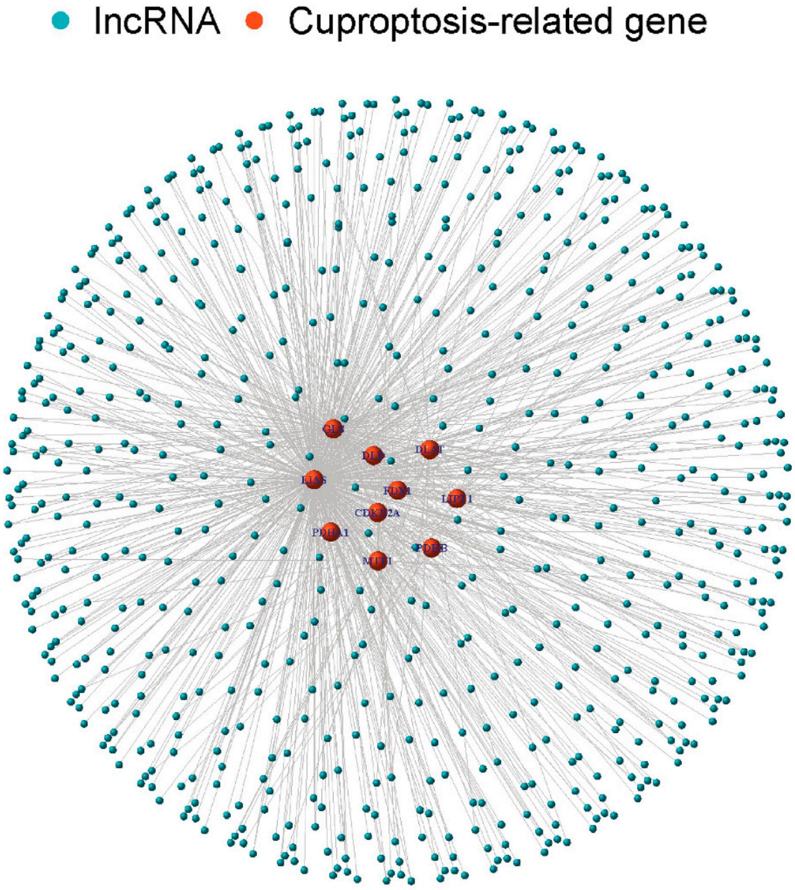

Figure S1. The network between cuproptosis-related genes and lncRNAs (correlation coefficients > 0.4 and p < 0.001).

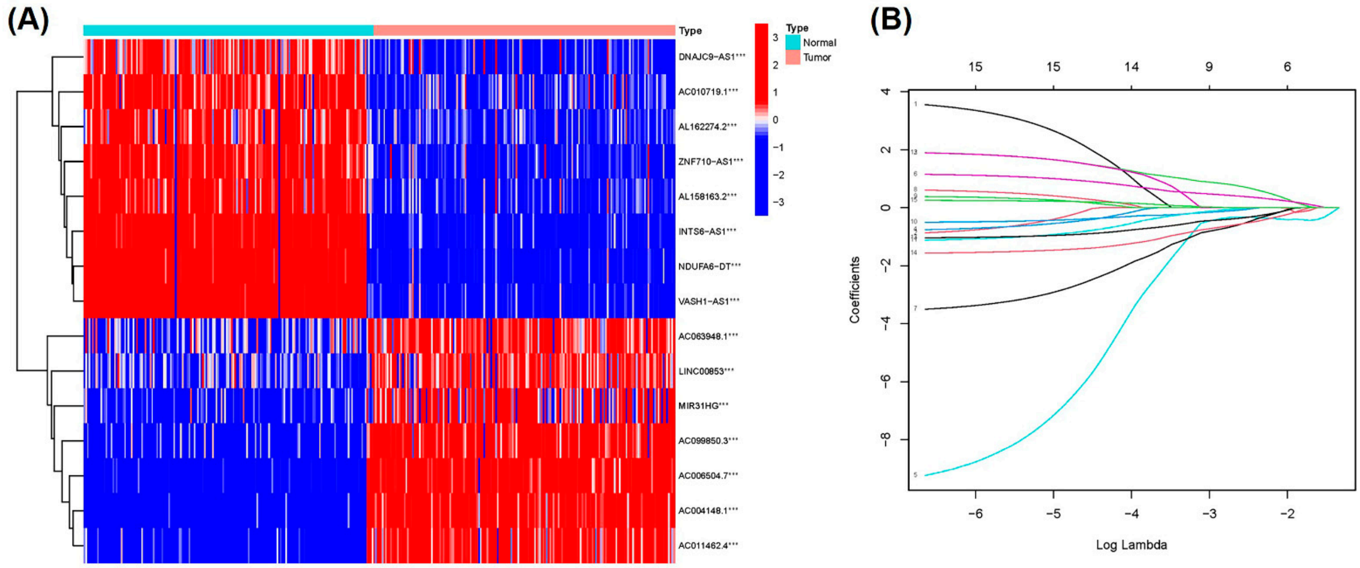

Figure S2. (A) The heatmap of the 15 differentially expressed cuproptosis-related lncRNA with prognostic value.**(B)** Distribution of LASSO coefficients. *** $p < 0.001$.

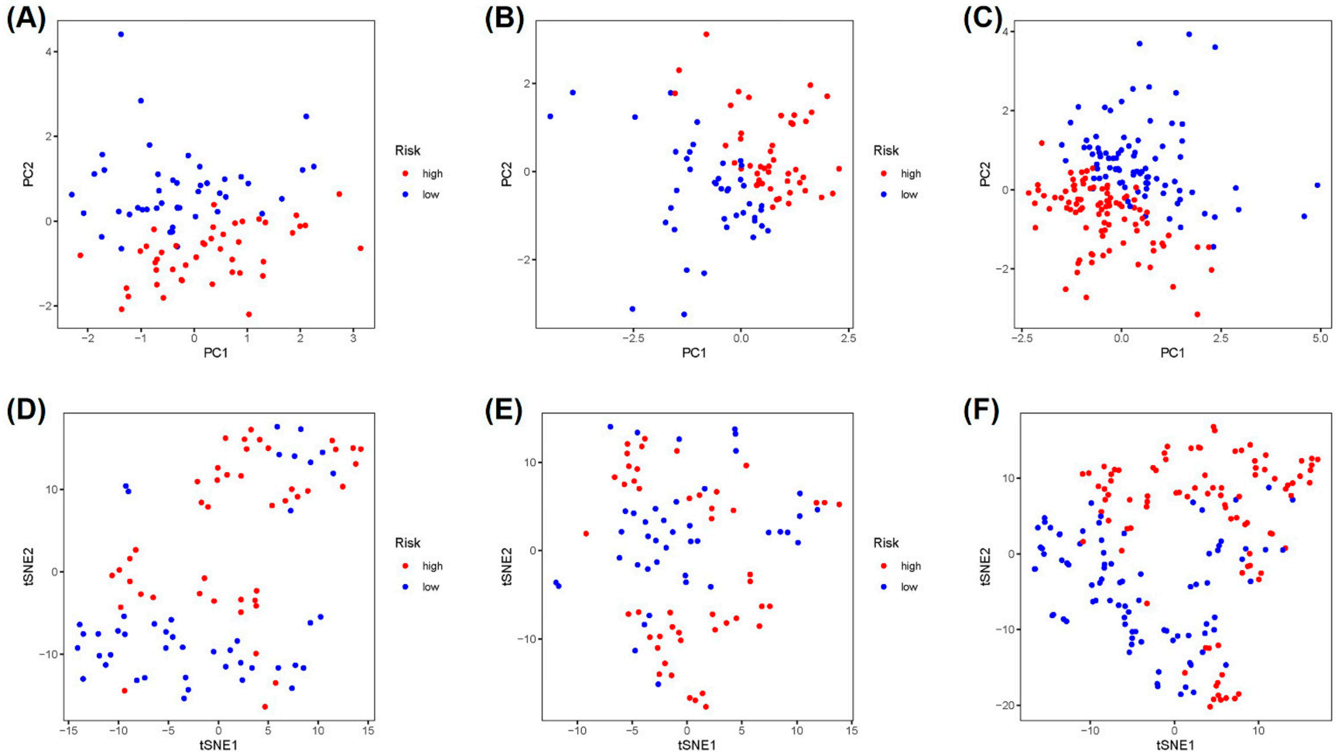

Figure S3. PCA and t-SNE scatter plot for the expression of 4 cuproptosis-related lncRNAs distinguishing the low- and high-risk cohorts. **(A and D)** the train cohort, **(B and E)** test cohort, and **(C and F)** entire cohort.

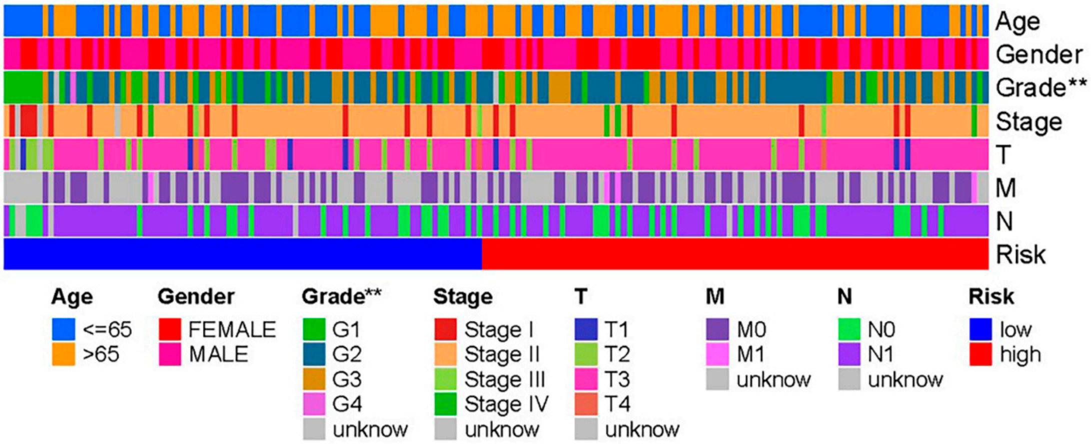

Figure S4. Strip chart showing relationships between clinical characteristics and cuproptosis-related lncRNA signature score.** $p < 0.01$.

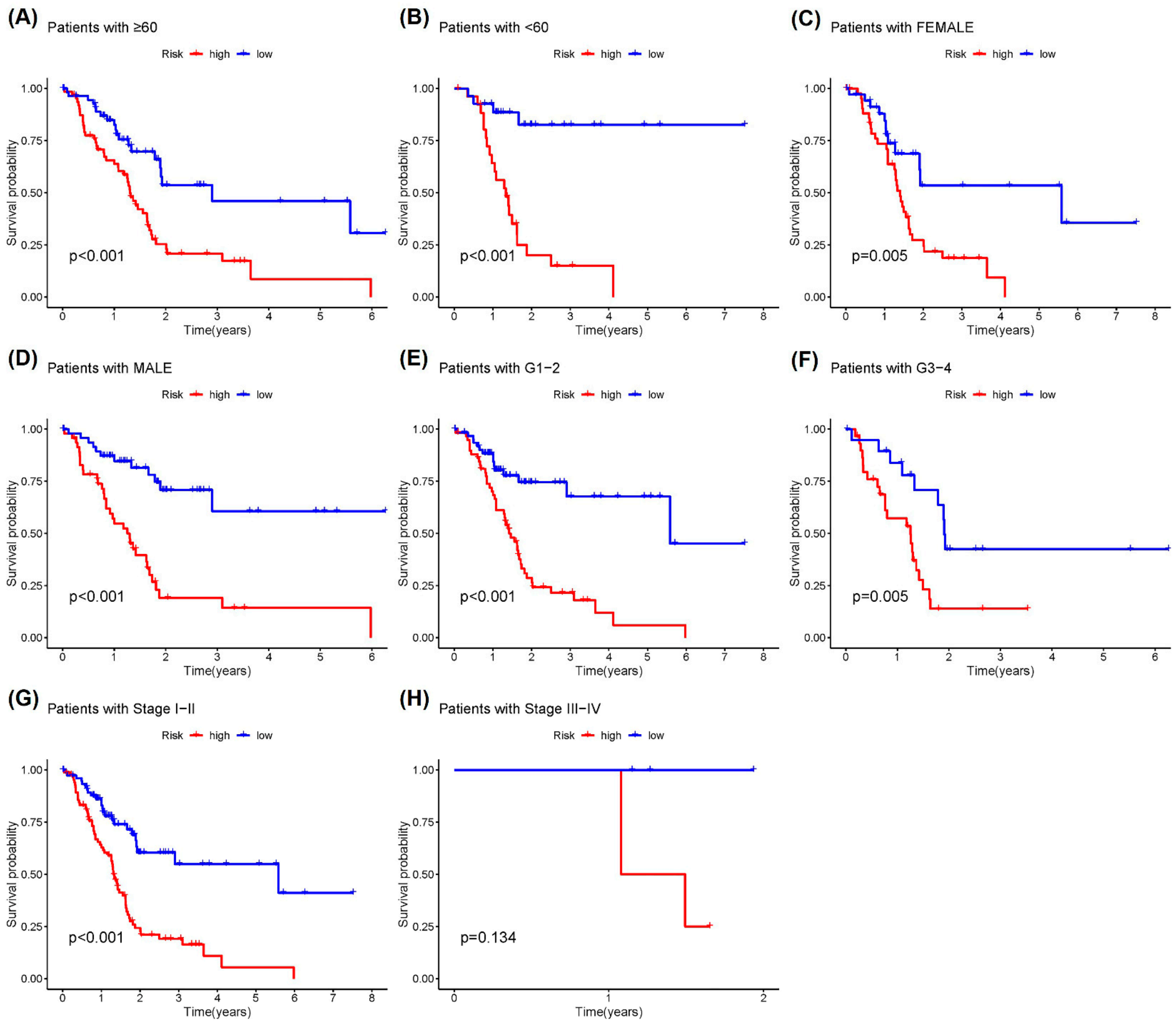

Figure S5. The subgroup analysis of overall survival of PAAD patients in the low- and high-risk groups by **(A and B)** age, **(C and D)** sex, **(E and F)** tumor grade, and **(G and H)** clinical stage.

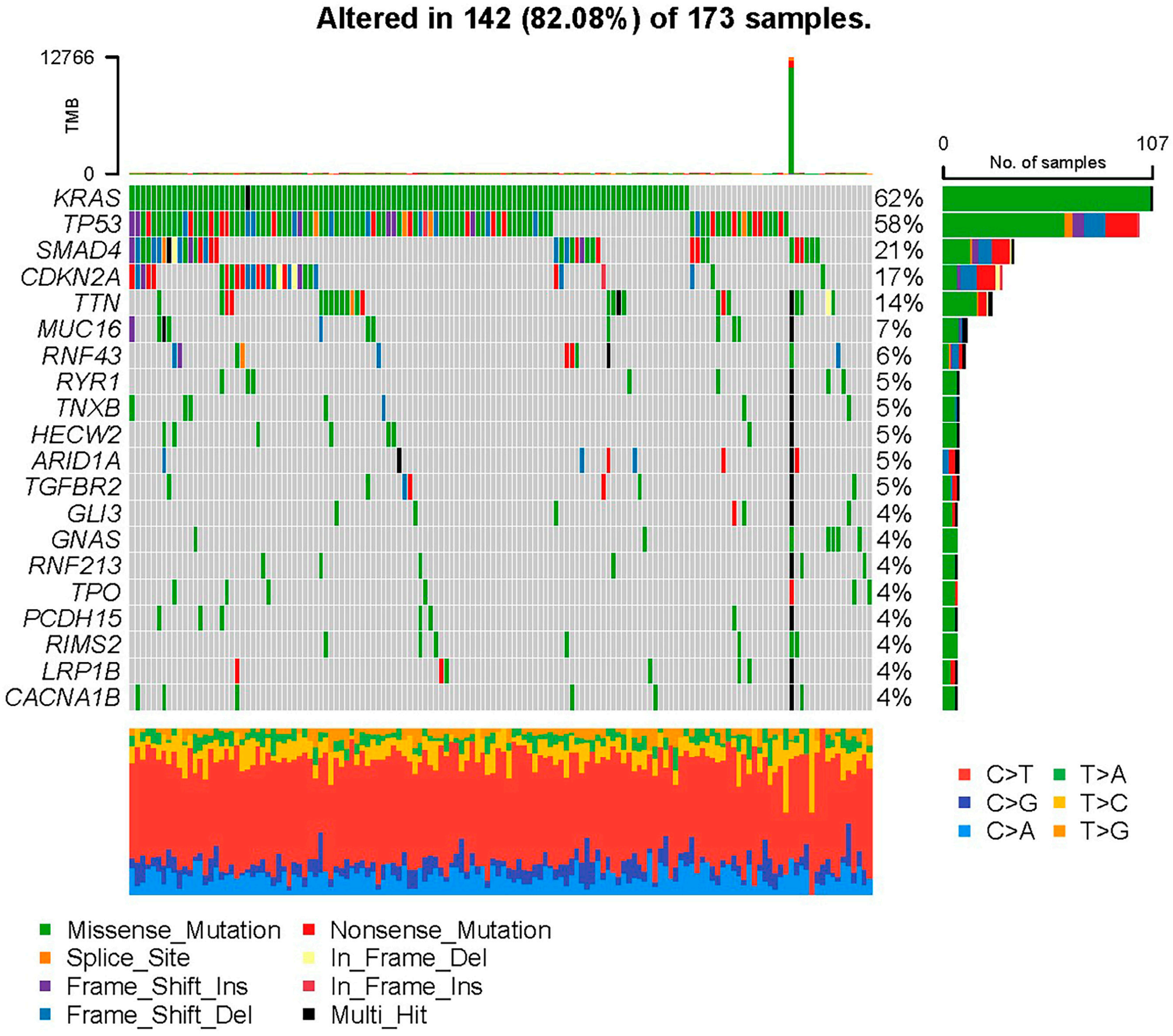

Figure S6. Waterfall plots of the 20 most frequently mutated genes in PAAD patients.

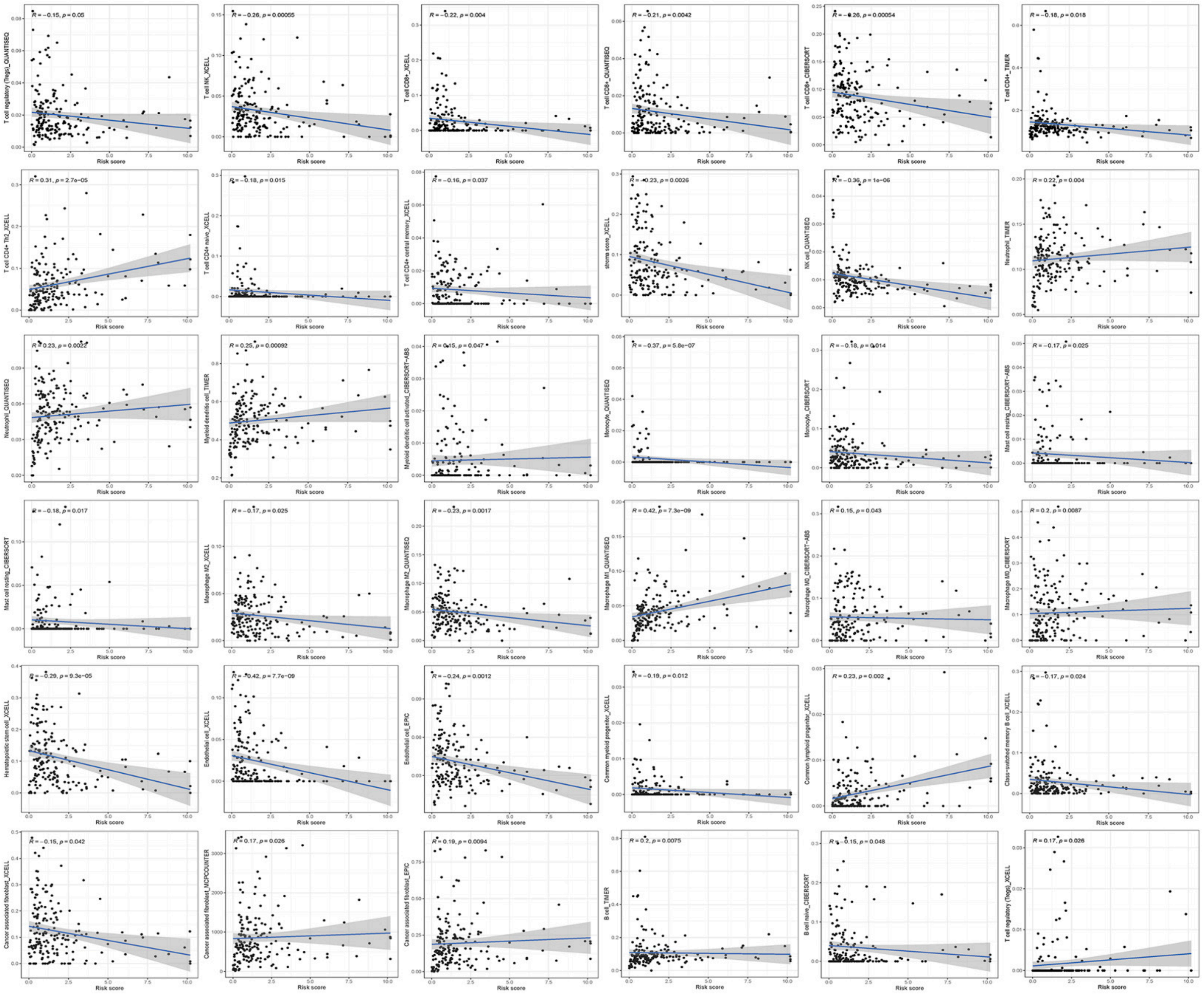

Figure S7. The correlation between risk score and immune cells.

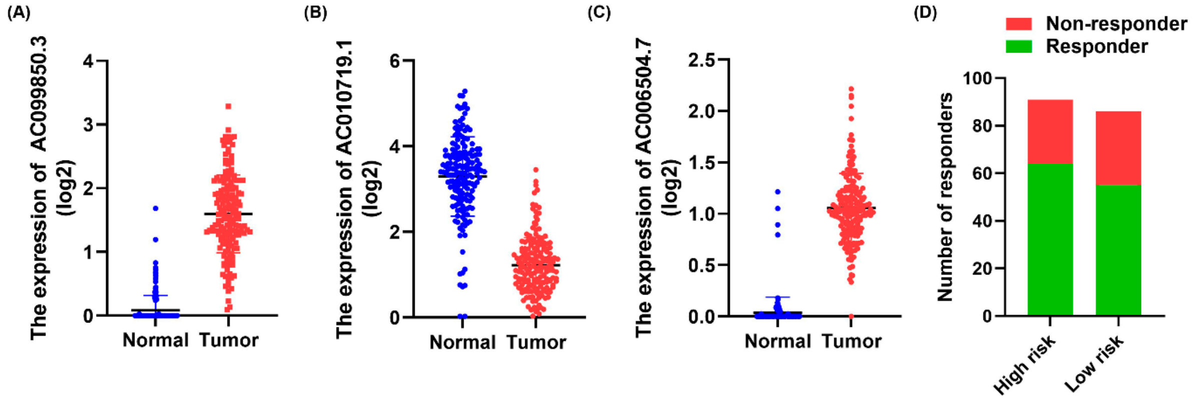

Figure S8. (A-C) The expression profile of lncRNAs in TCGA-PC dataset. **(D)** The difference in ICB response in TCGA-PAAD patients.
