# Supplementary material for: Systemic Analyses of Cuproptosis-Related lncRNAs in Pancreatic Adenocarcinoma, with a Focus on the Molecular Mechanism of LINC00853
Source: Int J Mol Sci. 2023 Apr 27;24(9):7923. doi: 10.3390/ijms24097923 (PMC10177970; doi:10.3390/ijms24097923)
Supplement: Supplementary file 1 [file ijms-24-07923-s001.zip › Supplementary Table S1.pdf]

Supplemental Table S1. The association between lncRNAs and cuproptosis regulate genes.

mRNA	lncRNA	Type
GLS	LINC02082	up
LIAS	AP000892.3	up
PDHA1	C1QTNF1-AS1	up
LIAS	AL731569.1	up
LIAS	AC087741.2	up
GLS	AL158055.1	up
LIAS	AC104785.1	up
MTF1	LINC02384	up
LIAS	AL033527.3	up
PDHB	MIR100HG	up
LIAS	AC092803.1	up
LIAS	AC108471.2	up
LIAS	AC093297.2	up
LIAS	AL023803.1	up
LIAS	MCF2L-AS1	up
PDHA1	MCF2L-AS1	up
LIAS	AL136531.1	up
LIAS	STXBP5-AS1	up
LIAS	AC005920.1	up
MTF1	PINK1-AS	up
LIAS	AC008608.2	up
LIAS	KCNJ2-AS1	up
LIAS	AC026367.1	up
GLS	AC026367.1	up
LIAS	AC027575.2	up
LIAS	AC124016.1	up
LIPT1	AC124016.1	up
LIAS	AL161772.1	up
DLAT	AC022034.1	up
LIAS	MINCR	up
LIAS	C9orf147	up
GLS	C9orf147	up
GLS	AC008050.1	up
LIAS	AC034198.2	up
LIAS	AC009686.2	up
LIAS	SCOC-AS1	up
DLD	AC005586.1	up
LIAS	AL133353.1	up
GLS	ST3GAL5-AS1	up

LIAS	AC007114.1	up
GLS	LINC01963	up
LIAS	AC106795.2	up
GLS	AC106795.2	up
LIAS	LINC00412	up
LIAS	ATP2B1-AS1	up
LIAS	AC010913.1	up
DLAT	RASAL2-AS1	up
LIAS	SEPSECS-AS1	up
GLS	SEPSECS-AS1	up
DLD	AL357054.4	up
LIAS	AC114271.1	up
LIAS	AC106052.1	up
LIAS	AL158163.2	up
LIAS	MAFG-DT	up
DLAT	AF127577.4	up
GLS	AF127577.4	up
LIAS	RN7SL832P	up
LIAS	DLEU1	up
LIAS	AL358781.2	up
GLS	AL359532.1	up
LIAS	AL050341.2	up
LIAS	AC097505.1	up
LIAS	AL035420.3	up
LIAS	AC069224.1	up
GLS	LINC01783	up
LIAS	AC124242.1	up
GLS	AC124242.1	up
GLS	AL157871.6	up
LIAS	AC018766.1	up
LIAS	RGMB-AS1	up
GLS	PIK3CD-AS2	up
LIAS	INTS6-AS1	up
GLS	TRAF3IP2-AS1	up
GLS	AL117335.1	up
GLS	ST20-AS1	up
LIAS	TMEM99	up
LIAS	AC006942.1	up
GLS	FLG-AS1	up
LIAS	AC011468.5	up
LIAS	AC025162.2	up
LIAS	AL391807.1	up
LIAS	STX18-AS1	up
LIAS	AL163051.1	up

LIAS	CEP83-DT	up
GLS	CEP83-DT	up
LIAS	AC073896.3	up
GLS	AC073896.3	up
LIAS	MNX1-AS1	up
LIAS	CASC2	up
GLS	CASC2	up
LIAS	CKMT2-AS1	up
GLS	CKMT2-AS1	up
GLS	AC145207.8	up
LIAS	AC007686.3	up
LIAS	ZSCAN16-AS1	up
LIAS	TMEM161B-AS1	up
LIPT1	LINC01569	up
LIAS	AL138762.1	up
LIAS	EXOC3-AS1	up
GLS	EXOC3-AS1	up
GLS	LINC01942	up
GLS	SDCBP2-AS1	up
LIAS	AC092171.3	up
LIAS	LINC02447	up
LIAS	HNF1A-AS1	up
LIAS	AC087500.1	up
LIAS	AC110609.1	up
LIAS	AC004812.2	up
LIAS	AL451069.3	up
LIAS	MESTIT1	up
LIAS	AL445645.1	up
LIAS	AC016394.1	up
LIAS	AL136040.1	up
CDKN2A	MIRLET7BHG	up
LIPT1	AC124798.1	up
LIPT1	LINC00853	up
LIAS	LINC00847	up
LIAS	ZNF213-AS1	up
LIAS	ZNF426-DT	up
PDHB	BOLA3-AS1	up
LIAS	UCKL1-AS1	up
LIAS	STK24-AS1	up
LIAS	AC096733.2	up
GLS	AC096733.2	up
LIAS	AC022916.1	up
LIAS	MAN1B1-DT	up
LIAS	STAM-AS1	up

LIAS	AC004477.1	up
LIAS	AC010327.5	up
LIAS	TMPO-AS1	up
GLS	AC127070.1	up
LIAS	LINC01144	up
LIAS	AC109347.1	up
LIAS	LINC00641	up
FDX1	MKLN1-AS	up
LIAS	AC118553.1	up
LIAS	AC145207.5	up
PDHA1	AP001542.3	up
LIAS	AL451064.1	up
LIAS	DICER1-AS1	up
PDHB	AC098484.1	up
LIAS	AC023632.2	up
GLS	AC023632.2	up
LIAS	C5orf38	up
GLS	C5orf38	up
LIAS	SNHG8	up
LIAS	COA6-AS1	up
LIAS	AC020558.2	up
GLS	AC020558.2	up
LIAS	AP003721.4	up
GLS	AP003721.4	up
DLAT	SUCLG2-AS1	up
MTF1	SUCLG2-AS1	up
GLS	AC104564.3	up
LIAS	AC074032.1	up
GLS	LINC01933	up
GLS	SOCS2-AS1	up
LIAS	ZNF337-AS1	up
LIAS	ATP6V0E2-AS1	up
GLS	ATP6V0E2-AS1	up
LIAS	AC119403.1	up
LIAS	AC008966.1	up
LIAS	AC104187.1	up
LIAS	AC064807.1	up
LIAS	AC100810.1	up
LIAS	AC005280.1	up
GLS	AC005280.1	up
LIAS	AL136295.7	up
GLS	NORAD	up
GLS	LINC01765	up
LIAS	AC138696.2	up

LIAS	SAPCD1-AS1	up
GLS	SAPCD1-AS1	up
LIAS	ARRDC1-AS1	up
LIAS	AC011477.2	up
LIAS	AC063948.1	up
GLS	LINC01252	up
GLS	LAMA5-AS1	up
LIAS	SNHG7	up
LIAS	LINC01003	up
LIAS	TDRKH-AS1	up
LIAS	POLR2J4	up
LIAS	EPB41L4A-AS1	up
LIAS	AL161421.1	up
GLS	AL161421.1	up
FDX1	AC005828.4	up
LIAS	AL138767.3	up
GLS	AL138767.3	up
LIAS	UGDH-AS1	up
GLS	ZNF667-AS1	up
LIAS	AL604028.1	up
LIAS	AC008438.1	up
GLS	LINC00271	up
LIAS	AL606970.1	up
GLS	AL606970.1	up
LIAS	TSC22D1-AS1	up
GLS	AP001625.2	up
LIAS	AC008105.1	up
GLS	AL354811.1	up
LIAS	CRTC3-AS1	up
LIAS	FAM218A	up
GLS	FAM218A	up
LIAS	LINC01816	up
LIAS	AL157813.1	up
GLS	AC079760.1	up
LIAS	LINC01522	up
GLS	LINC01522	up
LIAS	AC132938.1	up
LIAS	AC005076.1	up
LIAS	AC129507.3	up
LIAS	AL160314.2	up
LIAS	AC107982.3	up
LIAS	AC010997.4	up
MTF1	LINC00923	up
LIAS	AL135925.1	up

LIAS	AL355001.2	up
LIAS	MIS18A-AS1	up
LIAS	AC019131.2	up
LIAS	AC083880.1	up
LIAS	AL008723.2	up
GLS	AL008723.2	up
LIAS	LINC02321	up
GLS	LINC02321	up
LIAS	AP002360.1	up
LIAS	AC007292.1	up
FDX1	AC006237.1	up
LIAS	AC005062.1	up
LIAS	AC012313.5	up
GLS	AC012313.5	up
GLS	AC106795.5	up
GLS	LINC02256	up
LIAS	AL118558.3	up
LIAS	AC012213.4	up
GLS	AC012213.4	up
LIAS	AC104985.1	up
GLS	AC104985.1	up
LIAS	AC005225.3	up
MTF1	AC022509.3	up
GLS	AL139384.2	up
LIAS	AC068338.2	up
LIAS	CBR3-AS1	up
LIAS	ZNF460-AS1	up
CDKN2A	AL359922.2	up
MTF1	AC125807.2	up
LIAS	AC107068.1	up
LIAS	AC079174.2	up
LIAS	AL139383.1	up
DLD	AL139383.1	up
GLS	AL139383.1	up
LIAS	AC008124.1	up
LIAS	ARIH2OS	up
LIAS	AL590705.1	up
LIAS	C9orf163	up
LIAS	DHRS4-AS1	up
MTF1	AL031985.3	up
GLS	AC245060.5	up
LIAS	AL121929.2	up
GLS	AL121929.2	up
LIAS	AC139887.2	up

LIAS	ZBED5-AS1	up
LIAS	AC005775.1	up
LIAS	AC019257.2	up
DLAT	AC078923.1	up
LIAS	AC012467.2	up
GLS	AL021368.2	up
GLS	AC106795.3	up
LIAS	ELOA-AS1	up
LIAS	AC097478.1	up
LIAS	ACVR2B-AS1	up
LIAS	AC009126.1	up
LIAS	AC012442.2	up
LIAS	PPP1R12A-AS1	up
DLD	PPP1R12A-AS1	up
LIAS	MIR200CHG	up
LIAS	AC015917.2	up
LIAS	AL162377.1	up
GLS	AL162377.1	up
DLAT	BAALC-AS1	up
LIAS	AC005962.1	up
LIAS	SNHG20	up
LIAS	AL589765.7	up
GLS	AL589765.7	up
LIAS	SNHG11	up
LIAS	DNAJC9-AS1	up
GLS	DNAJC9-AS1	up
MTF1	AC073046.1	up
DLAT	RHPN1-AS1	up
LIAS	AL133520.1	up
GLS	PAXBP1-AS1	up
LIAS	AC004803.1	up
MTF1	AC004803.1	up
LIAS	AP001160.3	up
LIAS	AC005899.7	up
GLS	AC093227.1	up
LIAS	AL020996.1	up
LIAS	AC008443.1	up
DLAT	AC099850.3	up
LIAS	AC008393.1	up
LIAS	AC079766.1	up
LIAS	AC008115.4	up
GLS	AC008115.4	up
GLS	LINC01554	up
LIAS	NDUFA6-DT	up

LIAS	FAM66C	up
GLS	FAM66C	up
LIAS	AC025181.2	up
PDHA1	AC025181.2	up
LIAS	TUSC8	up
LIAS	LINC01607	up
LIAS	AC072061.1	up
LIAS	AC008014.1	up
GLS	AC010226.1	up
LIAS	AC008035.1	up
LIAS	AC121338.2	up
GLS	AC121338.2	up
LIAS	BSN-DT	up
FDX1	AC100797.1	up
LIAS	AC095057.3	up
LIAS	AC002470.1	up
LIAS	AC024575.1	up
LIAS	AL121917.1	up
GLS	AC104117.3	up
LIAS	AC006449.6	up
LIAS	FAM27E3	up
LIAS	AC107952.2	up
GLS	AC138649.1	up
LIAS	STIM2-AS1	up
LIAS	AC008105.2	up
LIAS	SNHG6	up
GLS	SNHG6	up
LIAS	AP001412.1	up
LIAS	MAPKAPK5-AS1	up
LIAS	AC002511.1	up
GLS	AC002511.1	up
LIAS	AC156455.1	up
LIAS	AC099684.2	up
GLS	AC099684.2	up
LIAS	Z97653.1	up
LIAS	TRAM2-AS1	up
LIAS	AL132639.2	up
MTF1	AC115837.1	up
GLS	AL121917.2	up
LIAS	Z95115.1	up
LIAS	CNNM3-DT	up
MTF1	AC018752.1	up
MTF1	AL133523.1	up
LIAS	TRIM52-AS1	up

GLS	TRIM52-AS1	up
LIAS	LINC00957	up
LIPT1	AC145285.2	up
DLAT	LINC01376	up
MTF1	AC000403.1	up
LIAS	AC099778.1	up
GLS	RFX3-AS1	up
LIPT1	AL590096.1	up
CDKN2A	CDKN2B-AS1	up
LIAS	AC002451.1	up
LIAS	AC018413.1	up
LIAS	AC087752.4	up
LIAS	AC008669.1	up
GLS	AC008669.1	up
LIAS	LIFR-AS1	up
LIAS	AC025175.1	up
GLS	AC025175.1	up
LIAS	AC109322.1	up
LIAS	AC087500.2	up
LIAS	RNASEH1-AS1	up
GLS	ADORA2A-AS1	up
DLD	AL356489.2	up
GLS	AL356489.2	up
LIAS	AC087521.2	up
LIAS	AL035071.1	up
GLS	AL035071.1	up
LIAS	MIR7-3HG	up
GLS	MIR7-3HG	up
LIAS	AL121832.2	up
LIAS	ILF3-DT	up
LIAS	AL596202.1	up
LIAS	AC127024.6	up
LIAS	AL035701.1	up
GLS	AL035701.1	up
LIAS	AC010300.1	up
LIAS	ST7-AS1	up
LIAS	AC093752.1	up
GLS	AC093752.1	up
LIAS	AL354696.2	up
LIAS	AC005229.4	up
GLS	AL049838.1	up
LIAS	AC073508.3	up
LIAS	AL135999.3	up
GLS	LINC01018	up

LIAS	RBM26-AS1	up
GLS	RBM26-AS1	up
LIAS	LINC01637	up
LIAS	AC116913.1	up
LIAS	AC011477.3	up
GLS	AC011477.3	up
LIAS	ENTPD3-AS1	up
LIPT1	AC083799.1	up
LIAS	AC020910.5	up
LIAS	AL162411.1	up
GLS	AL162411.1	up
LIAS	AC022306.3	up
GLS	DDN-AS1	up
LIAS	TP53TG1	up
LIAS	AC139530.1	up
LIAS	STPG3-AS1	up
LIAS	MORF4L2-AS1	up
LIAS	NNT-AS1	up
LIAS	NEBL-AS1	up
LIAS	AC010491.1	up
LIAS	AP000757.1	up
LIAS	LINC01006	up
LIAS	PART1	up
GLS	PART1	up
LIAS	ZNF571-AS1	up
GLS	AC087854.1	up
LIAS	AC018521.5	up
LIAS	AC092354.1	up
LIAS	LINC00909	up
LIAS	AC012213.1	up
GLS	AC012213.1	up
LIAS	AL359232.1	up
MTF1	LINC00940	up
LIAS	AC036176.1	up
LIAS	GLIDR	up
GLS	BX293535.1	up
LIAS	BCDIN3D-AS1	up
GLS	BCDIN3D-AS1	up
LIAS	AC079414.3	up
LIAS	GEMIN7-AS1	up
PDHB	ZNF710-AS1	up
GLS	AL022332.1	up
LIAS	AC125257.1	up
GLS	AC125257.1	up

MTF1	AC006064.2	up
LIAS	Z93930.2	up
LIAS	AC121761.2	up
LIAS	NIFK-AS1	up
LIAS	AC120053.1	up
GLS	LINC01146	up
GLS	AC233976.1	up
LIAS	LINC01534	up
LIAS	AL162171.1	up
GLS	AL162171.1	up
LIAS	BX537318.1	up
GLS	AL133325.3	up
LIAS	AL512625.1	up
LIAS	AL354920.1	up
GLS	GRPEL2-AS1	up
LIAS	AL358472.2	up
GLS	AC006369.1	up
LIAS	KIF9-AS1	up
LIAS	LINC01091	up
LIAS	CDC37L1-DT	up
LIAS	LINC00653	up
DLAT	LINC00630	up
MTF1	LINC00630	up
FDX1	AC010636.1	up
LIAS	DNAJC27-AS1	up
LIAS	TRPC7-AS1	up
LIAS	AC087623.3	up
LIAS	AL139289.2	up
LIAS	AC009403.1	up
LIAS	AC026691.1	up
LIAS	LINC02482	up
MTF1	AC073254.1	up
LIAS	AC004832.1	up
GLS	AC004832.1	up
LIAS	AF129075.2	up
LIAS	AL359513.1	up
LIAS	VASH1-AS1	up
LIAS	AC097641.2	up
LIAS	LIPE-AS1	up
LIAS	AL121583.1	up
GLS	AL121583.1	up
GLS	ZBED3-AS1	up
LIAS	AC000068.1	up
GLS	AC000068.1	up

LIAS	PXN-AS1	up
GLS	AL161804.1	up
LIAS	AC091153.3	up
LIAS	AL023806.1	up
GLS	AL023806.1	up
LIAS	AL645924.1	up
LIAS	AC120498.10	up
LIAS	AL031005.1	up
LIPT1	AP001830.1	up
LIAS	ZNF433-AS1	up
LIAS	APTR	up
LIAS	AP000894.4	up
GLS	AC011445.1	up
LIAS	AC064836.3	up
LIAS	RPARP-AS1	up
MTF1	OGFRP1	up
LIAS	AL161729.4	up
LIAS	AL138966.2	up
LIAS	AP001486.2	up
LIAS	AC026979.2	up
LIAS	AC087752.3	up
LIAS	HCG14	up
GLS	AC002511.2	up
GLS	AC005540.1	up
LIAS	AL589739.1	up
FDX1	AC123595.1	up
LIAS	AL391684.1	up
PDHA1	AC010719.1	up
LIAS	AC005034.4	up
GLS	AC005034.4	up
LIAS	AC092354.2	up
LIAS	AC017100.1	up
GLS	FAM182B	up
LIAS	SLC12A5-AS1	up
GLS	SLC12A5-AS1	up
LIAS	AC011477.1	up
LIAS	LINC00467	up
LIAS	Z97055.2	up
GLS	AC006213.5	up
LIAS	AC009812.1	up
LIAS	SEC24B-AS1	up
LIAS	AC096677.1	up
LIAS	AC048382.5	up
LIAS	AC000068.2	up

PDHA1	AC005083.1	up
LIAS	AC015961.2	up
LIAS	AC022893.2	up
GLS	AC022893.2	up
GLS	AC067852.3	up
GLS	AC127070.2	up
FDX1	AC004825.2	up
LIAS	AC022211.2	up
GLS	AC022211.2	up
LIAS	AC079305.1	up
LIAS	AL356740.1	up
PDHA1	AL356740.1	up
LIAS	AC090061.1	up
GLS	AC090061.1	up
LIAS	GIHCG	up
LIAS	TMEM44-AS1	up
LIAS	CCDC13-AS1	up
GLS	CCDC13-AS1	up
LIAS	LINC01750	up
GLS	AL359715.3	up
GLS	LINC02106	up
GLS	LINC01128	up
LIAS	GNAS-AS1	up
GLS	GNAS-AS1	up
LIAS	AC027644.3	up
PDHA1	RAB11B-AS1	up
LIAS	AP000350.5	up
GLS	AL049796.1	up
LIAS	AC004241.3	up
GLS	MAGI2-AS3	up
LIAS	AP003559.1	up
GLS	AP003559.1	up
GLS	AC079949.2	up
GLS	AC243830.2	up
LIAS	LINC01484	up
GLS	LINC01484	up
LIAS	AL136295.6	up
MTF1	AC005562.1	up
LIAS	LINC01431	up
DLAT	LY6E-DT	up
MTF1	AL731577.2	up
LIAS	AC021242.3	up
GLS	AC021242.3	up
PDHB	AC012085.2	up

GLS	AL359715.2	up
LIAS	AC024361.2	up
LIAS	AC129507.2	up
LIAS	VLDLR-AS1	up
LIAS	AC010624.1	up
LIAS	AC007066.2	up
LIAS	EDRF1-DT	up
GLS	COX10-AS1	up
LIAS	SRP14-AS1	up
MTF1	LINC00668	up
LIAS	DANCR	up
PDHA1	DANCR	up
LIAS	AC005498.2	up
LIAS	AC092171.5	up
GLS	SNHG14	up
FDX1	GRTP1-AS1	up
LIPT1	AC009148.1	up
LIAS	AC068338.3	up
LIAS	EIF2AK3-DT	up
GLS	AC022400.1	up
LIAS	AL596244.1	up
LIAS	AC127024.5	up
LIAS	AC024075.2	up
LIAS	AC006538.1	up
LIAS	AL355472.3	up
LIAS	AC040169.1	up
GLS	AC007406.3	up
LIAS	STX17-AS1	up
GLS	AL121672.2	up
LIAS	URB1-AS1	up
LIAS	AL355312.3	up
LIAS	MALINC1	up
LIAS	AL021368.3	up
GLS	AL021368.3	up
LIAS	SREBF2-AS1	up
GLS	AL390208.1	up
LIAS	AL137779.2	up
LIAS	AC064807.4	up
FDX1	AC006001.2	up
LIAS	AL732314.6	up
LIAS	AL136304.1	up
LIAS	LINC00663	up
LIAS	AC022098.3	up
LIAS	LINC01370	up

GLS	LINC01370	up
GLS	AL137009.1	up
LIAS	AC023509.3	up
MTF1	AL139035.1	up
LIAS	AC108134.4	up
LIPT1	AC090527.3	up
LIAS	AC002467.1	up
PDHB	AP001107.5	up
LIAS	AC138207.2	up
LIAS	AC068473.5	up
LIAS	RUNDC3A-AS1	up
MTF1	AC234775.3	up
CDKN2A	AC138150.2	up
LIAS	AC092119.3	up
GLS	AC092119.3	up
GLS	AC092818.1	up
LIAS	AL354892.2	up
LIAS	AC006504.7	up
DLD	RAP2C-AS1	up
MTF1	RAP2C-AS1	up
LIAS	AL133343.2	up
GLS	AL133343.2	up
LIAS	SCAMP1-AS1	up
GLS	SCAMP1-AS1	up
LIAS	AC133552.5	up
LIAS	THAP7-AS1	up
LIAS	FRG1-DT	up
LIAS	AC092574.1	up
GLS	AC092574.1	up
LIAS	LINC00240	up
LIAS	AC012555.1	up
GLS	AC012555.1	up
LIAS	OLMALINC	up
GLS	OLMALINC	up
LIAS	AC021087.1	up
GLS	AC021087.1	up
LIAS	MIR4458HG	up
LIAS	EXTL3-AS1	up
GLS	EXTL3-AS1	up
LIAS	AP001267.3	up
LIAS	FGF14-AS2	up
PDHB	MBNL1-AS1	up
FDX1	MIR202HG	up
GLS	AC008808.2	up

CDKN2A	MIR31HG	up
LIAS	LINC00476	up
GLS	LINC01197	up
LIAS	FOXP4-AS1	up
LIAS	PITPNA-AS1	up
FDX1	AC093110.1	up
LIAS	IL6R-AS1	up
GLS	IL6R-AS1	up
GLS	SNTG2-AS1	up
LIAS	AC034236.2	up
LIAS	AC005696.1	up
PDHA1	AC005696.1	up
LIAS	OSER1-DT	up
LIAS	AL450998.2	up
LIAS	AC107375.1	up
LIAS	MAST4-AS1	up
LIAS	AC005089.1	up
GLS	AC005089.1	up
LIAS	C1orf229	up
DLAT	USP2-AS1	up
LIAS	AL136162.1	up
MTF1	AC091182.1	up
LIAS	LINC01315	up
LIAS	AL122008.3	up
LIAS	CEBPB-AS1	up
LIAS	AC011444.1	up
LIAS	OTUD6B-AS1	up
GLS	OTUD6B-AS1	up
GLS	MCPH1-AS1	up
LIAS	KMT2E-AS1	up
LIAS	USP27X-AS1	up
MTF1	AC104411.1	up
LIAS	AL356512.1	up
LIAS	AL589765.1	up
GLS	AL589765.1	up
GLS	AC079949.1	up
LIAS	ZNF793-AS1	up
GLS	ZNF793-AS1	up
FDX1	AC078880.3	up
LIAS	AC004982.1	up
LIAS	HDHD5-AS1	up
LIAS	AL132657.1	up
LIPT1	AL592435.1	up
LIAS	AL356019.2	up

GLS	AL356019.2	up
LIAS	AC005911.1	up
LIAS	AC022079.2	up
GLS	ZNF582-AS1	up
LIAS	TTC28-AS1	up
GLS	AC124312.3	up
FDX1	LINC02038	up
GLS	AL158212.3	up
LIAS	AC009779.2	up
LIAS	SLC25A5-AS1	up
GLS	SLC25A5-AS1	up
LIAS	SGMS1-AS1	up
LIAS	AL118558.4	up
LIAS	AC097468.1	up
GLS	AC097468.1	up
LIAS	ALOX12-AS1	up
LIAS	AC125494.2	up
LIAS	ERVK9-11	up
GLS	ERVK9-11	up
PDHA1	AL731684.1	up
LIAS	AL133325.2	up
GLS	AL133325.2	up
LIAS	PSMG3-AS1	up
LIAS	AC009118.3	up
LIAS	UBA6-AS1	up
MTF1	LINC01521	up
DLAT	AL450384.2	down
DLAT	AL096870.2	down
DLAT	AC135050.6	down
DLAT	AC084824.5	down
DLAT	AC245884.8	down
DLAT	ZNF213-AS1	down
DLD	MIR4435-2HG	down
DLAT	AC005387.1	down
DLAT	AC004846.2	down
PDHB	AL031714.1	down
DLAT	AL645940.1	down
LIAS	AC009093.1	down
MTF1	AL157392.4	down
DLAT	AC005899.6	down
DLAT	AL136295.7	down
DLAT	AC011477.2	down
DLAT	PTOV1-AS2	down
MTF1	AC104463.2	down

DLAT	SEMA3F-AS1	down
DLAT	ZKSCAN2-DT	down
MTF1	ZKSCAN2-DT	down
DLAT	GUSBP11	down
DLD	LINC01138	down
PDHB	AC137932.3	down
MTF1	AP003352.1	down
LIAS	CASC15	down
DLAT	AC012615.1	down
PDHB	AC009133.1	down
MTF1	SNHG12	down
DLAT	AC004148.1	down
LIAS	AC015712.2	down
DLD	MIR222HG	down
DLAT	AC011462.4	down
MTF1	AC011462.4	down
DLAT	AL049840.5	down
DLAT	AC005306.1	down
DLAT	AL162274.2	down
MTF1	AC103706.1	down
DLAT	AP001062.1	down
GLS	LBX2-AS1	down
MTF1	AC093788.1	down
MTF1	AL021707.8	down
DLAT	AC009283.1	down
MTF1	AC009283.1	down
LIAS	AP001189.1	down
PDHA1	AP001189.1	down
DLD	AC010542.5	down
MTF1	AC010542.5	down
DLAT	AC087741.1	down
DLAT	AC024075.2	down
DLAT	AC016957.2	down
DLAT	U47924.3	down
DLAT	LINC01089	down
MTF1	AC005726.3	down
LIAS	AP001189.3	down
DLAT	Z69706.1	down
DLAT	LINC01176	down
DLAT	AL021707.6	down
MTF1	AL021707.6	down
