# Supplementary material for: Systemic Analyses of Cuproptosis-Related lncRNAs in Pancreatic Adenocarcinoma, with a Focus on the Molecular Mechanism of LINC00853
Source: Int J Mol Sci. 2023 Apr 27;24(9):7923. doi: 10.3390/ijms24097923 (PMC10177970; doi:10.3390/ijms24097923)
Supplement: Supplementary file 1 [file ijms-24-07923-s001.zip › Supplementary Table S2.pdf]

Supplemental Table S2. High- and low risk patient categories.

id	risk	id	risk
TCGA-IB-7888	high	TCGA-2J-AABO	low
TCGA-FB-A5VM	high	TCGA-H6-A45N	low
TCGA-HZ-8315	high	TCGA-XD-AAUH	low
TCGA-IB-7893	high	TCGA-HZ-8519	low
TCGA-M8-A5N4	high	TCGA-HV-AA8V	low
TCGA-HV-A7OL	high	TCGA-Q3-A5QY	low
TCGA-IB-7897	high	TCGA-3A-A9IL	low
TCGA-HZ-A77O	high	TCGA-3E-AAAY	low
TCGA-IB-A5SO	high	TCGA-FB-A4P6	low
TCGA-HZ-8638	high	TCGA-XN-A8T5	low
TCGA-L1-A7W4	high	TCGA-IB-8127	low
TCGA-IB-7886	high	TCGA-S4-A8RM	low
TCGA-3A-A9I9	high	TCGA-F2-6880	low
TCGA-2L-AAQJ	high	TCGA-IB-A5ST	low
TCGA-3A-A9IH	high	TCGA-2J-AABA	low
TCGA-HZ-7924	high	TCGA-Q3-AA2A	low
TCGA-HZ-7926	high	TCGA-LB-A9Q5	low
TCGA-HZ-A4BH	high	TCGA-HZ-A49G	low
TCGA-FB-AAQ2	high	TCGA-HZ-A49H	low
TCGA-3A-A9IC	high	TCGA-FB-AAPZ	low
TCGA-FB-AAQ6	high	TCGA-2J-AABF	low
TCGA-2J-AAB1	high	TCGA-HZ-8002	low
TCGA-3E-AAAZ	high	TCGA-HV-A5A6	low
TCGA-HZ-8637	high	TCGA-HZ-8001	low
TCGA-FB-AAPU	high	TCGA-HZ-7923	low
TCGA-HZ-7922	high	TCGA-HZ-7920	low
TCGA-2J-AABU	high	TCGA-IB-A6UF	low
TCGA-US-A77J	high	TCGA-HV-A5A5	low
TCGA-3A-A9J0	high	TCGA-HZ-7918	low
TCGA-2L-AAQL	high	TCGA-FB-AAPP	low
TCGA-IB-7887	high	TCGA-F2-A44H	low
TCGA-IB-A5SP	high	TCGA-IB-AAUW	low
TCGA-3A-A9IB	high	TCGA-3A-A9IS	low
TCGA-IB-7889	high	TCGA-IB-AAUT	low
TCGA-IB-AAUS	high	TCGA-Z5-AAPL	low
TCGA-F2-A44G	high	TCGA-IB-AAUR	low
TCGA-IB-7890	high	TCGA-FB-AAPS	low
TCGA-FB-A545	high	TCGA-2J-AAB4	low
TCGA-F2-A8YN	high	TCGA-IB-AAUV	low
TCGA-HZ-A49I	high	TCGA-RL-AAAS	low

TCGA-2L-AAQA	high	TCGA-XD-AAUI	low
TCGA-US-A77G	high	TCGA-3A-A9IJ	low
TCGA-IB-A5SQ	high	TCGA-FB-AAPY	low
TCGA-FB-AAPQ	high	TCGA-H8-A6C1	low
TCGA-FB-AAQ1	high	TCGA-YB-A89D	low
TCGA-2L-AAQE	high	TCGA-YH-A8SY	low
TCGA-HZ-8003	high	TCGA-2J-AABT	low
TCGA-IB-AAUU	high	TCGA-3A-A9IX	low
TCGA-PZ-A5RE	high	TCGA-3A-A9IV	low
TCGA-F2-6879	high	TCGA-3A-A9IR	low
TCGA-HZ-A9TJ	high	TCGA-H6-8124	low
TCGA-US-A77E	high	TCGA-LB-A8F3	low
TCGA-IB-7654	high	TCGA-YY-A8LH	low
TCGA-2J-AAB8	high	TCGA-FB-A78T	low
TCGA-HZ-8005	high	TCGA-3A-A9I5	low
TCGA-2J-AAB9	high	TCGA-XN-A8T3	low
TCGA-US-A779	high	TCGA-IB-AAUP	low
TCGA-HZ-7925	high	TCGA-HZ-8317	low
TCGA-FB-AAQ0	high	TCGA-RB-AA9M	low
TCGA-3A-A9IU	high	TCGA-3A-A9IO	low
TCGA-HV-AA8X	high	TCGA-2L-AAQM	low
TCGA-HZ-A77Q	high	TCGA-HZ-A8P0	low
TCGA-HZ-7289	high	TCGA-S4-A8RO	low
TCGA-2J-AABH	high	TCGA-S4-A8RP	low
TCGA-IB-7645	high	TCGA-IB-AAUM	low
TCGA-IB-7649	high	TCGA-HZ-A8P1	low
TCGA-IB-AAUN	high	TCGA-2J-AABV	low
TCGA-IB-A5SS	high	TCGA-HZ-A77P	low
TCGA-2J-AABI	high	TCGA-HV-A5A3	low
TCGA-IB-AAUO	high	TCGA-OE-A75W	low
TCGA-2J-AAB6	high	TCGA-3A-A9IN	low
TCGA-IB-7885	high	TCGA-F2-7276	low
TCGA-IB-7651	high	TCGA-2J-AABE	low
TCGA-IB-7646	high	TCGA-RB-A7B8	low
TCGA-3A-A9IZ	high	TCGA-IB-A6UG	low
TCGA-FB-A7DR	high	TCGA-IB-AAUQ	low
TCGA-2J-AABR	high	TCGA-3A-A9I7	low
TCGA-F2-A7TX	high	TCGA-XD-AAUG	low
TCGA-XD-AAUL	high	TCGA-HV-A7OP	low
TCGA-IB-7644	high	TCGA-US-A774	low
TCGA-HZ-7919	high	TCGA-IB-8126	low
TCGA-F2-7273	high	TCGA-FB-AAQ3	low
TCGA-2L-AAQI	high	TCGA-2J-AABK	low

TCGA-HZ-A4BK	high	TCGA-HV-A5A4	low
TCGA-US-A776	high	TCGA-FB-A4P5	low
TCGA-IB-A7M4	high	TCGA-2J-AABP	low
TCGA-LB-A7SX	high		
TCGA-IB-7652	high		
TCGA-HZ-8636	high		
TCGA-IB-A7LX	high		
TCGA-IB-7891	high		
