# Supplementary material for: Systemic Analyses of Cuproptosis-Related lncRNAs in Pancreatic Adenocarcinoma, with a Focus on the Molecular Mechanism of LINC00853
Source: Int J Mol Sci. 2023 Apr 27;24(9):7923. doi: 10.3390/ijms24097923 (PMC10177970; doi:10.3390/ijms24097923)
Supplement: Supplementary file 1 [file ijms-24-07923-s001.zip › Supplementary Table S3.pdf]

Supplemental Table S3. Spearman Correlation Results of Immune Invasion and Risk Score

immune	cor	pvalue
B cell_TIMER	0.200366	0.007496
T cell CD4+_TIMER	-0.17816	0.017669
Neutrophil_TIMER	0.215378	0.00399
Myeloid dendritic cell_TIMER	0.246961	0.00092
B cell naive_CIBERSORT	-0.149	0.047784
T cell CD8+_CIBERSORT	-0.25754	0.000539
Monocyte_CIBERSORT	-0.18468	0.01386
Macrophage M0_CIBERSORT	0.196608	0.008721
Mast cell resting_CIBERSORT	-0.17967	0.016712
Macrophage M0_CIBERSORT-ABS	0.151981	0.043446
Myeloid dendritic cell activated_CIBERSORT-ABS	0.14931	0.047311
Mast cell resting_CIBERSORT-ABS	-0.16793	0.025472
Macrophage M1_QUANTISEQ	0.417629	7.32E-09
Macrophage M2_QUANTISEQ	-0.23436	0.001691
Monocyte_QUANTISEQ	-0.36528	5.76E-07
Neutrophil_QUANTISEQ	0.228714	0.002199
NK cell_QUANTISEQ	-0.35763	1.02E-06
T cell CD8+_QUANTISEQ	-0.21414	0.00421
T cell regulatory (Tregs)_QUANTISEQ	-0.1477	0.049784
Cancer associated fibroblast_MCPCOUNTER	0.16713	0.026187
T cell CD4+ naive_XCELL	-0.1818	0.015445
T cell CD4+ central memory_XCELL	-0.15684	0.037088
T cell CD8+_XCELL	-0.21516	0.004027
Class-switched memory B cell_XCELL	-0.17006	0.023638
Common lymphoid progenitor_XCELL	0.230493	0.002026
Common myeloid progenitor_XCELL	-0.18881	0.011839
Endothelial cell_XCELL	-0.41702	7.74E-09
Cancer associated fibroblast_XCELL	-0.15287	0.042213
Hematopoietic stem cell_XCELL	-0.28945	9.32E-05
Macrophage M2_XCELL	-0.1689	0.024618
T cell NK_XCELL	-0.25724	0.000547
T cell CD4+ Th2_XCELL	0.309606	2.75E-05
T cell regulatory (Tregs)_XCELL	0.166933	0.026367
stroma score_XCELL	-0.22533	0.002566
Cancer associated fibroblast_EPIC	0.194624	0.009436
Endothelial cell_EPIC	-0.24142	0.001207
