# Supplementary material for: Systemic Analyses of Cuproptosis-Related lncRNAs in Pancreatic Adenocarcinoma, with a Focus on the Molecular Mechanism of LINC00853
Source: Int J Mol Sci. 2023 Apr 27;24(9):7923. doi: 10.3390/ijms24097923 (PMC10177970; doi:10.3390/ijms24097923)
Supplement: Supplementary file 1 [file ijms-24-07923-s001.zip › Supplementary Table S4.pdf]

Supplemental Table S4. Full names of the 10 cuproptosis related regulatory genes

Gene symbol	Gene name
FDX1	ferredoxin 1
LIAS	lipoyl syndrome
LIPT1	lipolytransferase 1
DLD	dihydrolipoamide dehydrogenase
DLAT	dihydrolipoamide s-acetyltransferase
PDHA1	pyruvate dehydrogenase E1 subunit alpha 1
PDHB	pyruvate dehydrogenase E1 subunit beta
MTF1	metal regulatory transcription factor 1
GLS	Glutaminase
CDKN2A	cyclin dependent kinase inhibitor 2A
