# Supplementary material for: Systemic Analyses of Cuproptosis-Related lncRNAs in Pancreatic Adenocarcinoma, with a Focus on the Molecular Mechanism of LINC00853
Source: Int J Mol Sci. 2023 Apr 27;24(9):7923. doi: 10.3390/ijms24097923 (PMC10177970; doi:10.3390/ijms24097923)
Supplement: Supplementary file 1 [file ijms-24-07923-s001.zip › Supplementary Table S5.pdf]

Supplemental Table S5. Train and test cohort patient grouping information.

Train cohort	Test cohort
TCGA-IB-7888	TCGA-FB-AAQ1
TCGA-2J-AABO	TCGA-2L-AAQE
TCGA-FB-A5VM	TCGA-HZ-8003
TCGA-HZ-8315	TCGA-YH-A8SY
TCGA-IB-7893	TCGA-2J-AABT
TCGA-M8-A5N4	TCGA-IB-AAUU
TCGA-HV-A7OL	TCGA-PZ-A5RE
TCGA-IB-7897	TCGA-3A-A9IX
TCGA-H6-A45N	TCGA-F2-6879
TCGA-XD-AAUH	TCGA-3A-A9IV
TCGA-HZ-A77O	TCGA-3A-A9IR
TCGA-IB-A5SO	TCGA-HZ-A9TJ
TCGA-HZ-8519	TCGA-H6-8124
TCGA-HV-AA8V	TCGA-LB-A8F3
TCGA-Q3-A5QY	TCGA-US-A77E
TCGA-3A-A9IL	TCGA-YY-A8LH
TCGA-3E-AAAY	TCGA-IB-7654
TCGA-FB-A4P6	TCGA-FB-A78T
TCGA-XN-A8T5	TCGA-2J-AAB8
TCGA-HZ-8638	TCGA-3A-A9I5
TCGA-L1-A7W4	TCGA-HZ-8005
TCGA-IB-7886	TCGA-2J-AAB9
TCGA-IB-8127	TCGA-US-A779
TCGA-S4-A8RM	TCGA-HZ-7925
TCGA-3A-A9I9	TCGA-XN-A8T3
TCGA-F2-6880	TCGA-FB-AAQ0
TCGA-2L-AAQJ	TCGA-IB-AAUP
TCGA-3A-A9IH	TCGA-HZ-8317
TCGA-IB-A5ST	TCGA-3A-A9IU
TCGA-HZ-7924	TCGA-HV-AA8X
TCGA-HZ-7926	TCGA-HZ-A77Q
TCGA-2J-AABA	TCGA-HZ-7289
TCGA-HZ-A4BH	TCGA-RB-AA9M
TCGA-Q3-AA2A	TCGA-3A-A9IO
TCGA-FB-AAQ2	TCGA-2J-AABH
TCGA-LB-A9Q5	TCGA-IB-7645
TCGA-3A-A9IC	TCGA-2L-AAQM
TCGA-HZ-A49G	TCGA-IB-7649

TCGA-HZ-A49H	TCGA-HZ-A8P0
TCGA-FB-AAPZ	TCGA-S4-A8RO
TCGA-2J-AABF	TCGA-IB-AAUN
TCGA-HZ-8002	TCGA-S4-A8RP
TCGA-FB-AAQ6	TCGA-IB-AAUM
TCGA-2J-AAB1	TCGA-HZ-A8P1
TCGA-HV-A5A6	TCGA-2J-AABV
TCGA-HZ-8001	TCGA-IB-A5SS
TCGA-3E-AAAZ	TCGA-2J-AABI
TCGA-HZ-7923	TCGA-IB-AAUO
TCGA-HZ-8637	TCGA-2J-AAB6
TCGA-HZ-7920	TCGA-IB-7885
TCGA-FB-AAPU	TCGA-IB-7651
TCGA-HZ-7922	TCGA-HZ-A77P
TCGA-IB-A6UF	TCGA-IB-7646
TCGA-HV-A5A5	TCGA-HV-A5A3
TCGA-2J-AABU	TCGA-OE-A75W
TCGA-US-A77J	TCGA-3A-A9IZ
TCGA-3A-A9J0	TCGA-3A-A9IN
TCGA-2L-AAQL	TCGA-F2-7276
TCGA-HZ-7918	TCGA-2J-AABE
TCGA-IB-7887	TCGA-RB-A7B8
TCGA-IB-A5SP	TCGA-IB-A6UG
TCGA-FB-AAPP	TCGA-IB-AAUQ
TCGA-F2-A44H	TCGA-FB-A7DR
TCGA-3A-A9IB	TCGA-2J-AABR
TCGA-IB-AAUW	TCGA-3A-A9I7
TCGA-3A-A9IS	TCGA-F2-A7TX
TCGA-IB-AAUT	TCGA-XD-AAUL
TCGA-Z5-AAPL	TCGA-IB-7644
TCGA-IB-7889	TCGA-HZ-7919
TCGA-IB-AAUR	TCGA-F2-7273
TCGA-FB-AAPS	TCGA-2L-AAQI
TCGA-IB-AAUS	TCGA-XD-AAUG
TCGA-2J-AAB4	TCGA-HZ-A4BK
TCGA-F2-A44G	TCGA-US-A776
TCGA-IB-AAUV	TCGA-IB-A7M4
TCGA-RL-AAAS	TCGA-LB-A7SX
TCGA-IB-7890	TCGA-HV-A7OP
TCGA-XD-AAUI	TCGA-IB-7652
TCGA-FB-A545	TCGA-US-A774
TCGA-3A-A9IJ	TCGA-IB-8126
TCGA-F2-A8YN	TCGA-FB-AAQ3

TCGA-HZ-A49I	TCGA-2J-AABK
TCGA-FB-AAPY	TCGA-HV-A5A4
TCGA-2L-AAQA	TCGA-HZ-8636
TCGA-H8-A6C1	TCGA-FB-A4P5
TCGA-US-A77G	TCGA-2J-AABP
TCGA-YB-A89D	TCGA-IB-A7LX
TCGA-IB-A5SQ	TCGA-IB-7891
TCGA-FB-AAPQ	
