# Supplementary material for: Systemic Analyses of Cuproptosis-Related lncRNAs in Pancreatic Adenocarcinoma, with a Focus on the Molecular Mechanism of LINC00853
Source: Int J Mol Sci. 2023 Apr 27;24(9):7923. doi: 10.3390/ijms24097923 (PMC10177970; doi:10.3390/ijms24097923)
Supplement: Supplementary file 1 [file ijms-24-07923-s001.zip › Supplementary Table S6.pdf]

Supplemental Table S6. The primers of selected cuproptosis-related lncRNAs.

Ensembl_ID	lncRNA	Sequence (5' - 3')
ENSG00000224805	LINC00853	Forward: AAAGGCTAGGCGATCCCACA
		Reverse: ACTCCCTAGCTTGGCTCTCCT
ENSG00000265415	AC099850.3	Forward: AGGGTCTCGCTATGTTTCCC
		Reverse: TCTCTGAAGTCCATAGCAGGTC
ENSG00000270933	AC010719.1	Forward: AGTGGAATGGAGGTTTGGGT
		Reverse: CCTTCCCTTACCTGTCAGCA
ENSG00000272635	AC006504.7	Forward: ACCTGGTCTGCGAGAAATCA
		Reverse: GCTTGCCCTTGGATCATCAG
